# Synthesis and Oxygen Mobility of Bismuth Cerates and Titanates with Pyrochlore Structure

**DOI:** 10.3390/membranes13060598

**Published:** 2023-06-13

**Authors:** Yuliya Bespalko, Nikita Eremeev, Ekaterina Sadovskaya, Tamara Krieger, Olga Bulavchenko, Evgenii Suprun, Mikhail Mikhailenko, Mikhail Korobeynikov, Vladislav Sadykov

**Affiliations:** 1 Federal Research Center, Boreskov Institute of Catalysis SB RAS, Novosibirsk, Akad. Lavrentieva Ave. 5, 630090 Novosibirsk, Russia; yeremeev21@catalysis.ru (N.E.); sadovsk@catalysis.ru (E.S.); krieger@catalysis.ru (T.K.); obulavchenko@catalysis.ru (O.B.); suprun@catalysis.ru (E.S.); sadykov@catalysis.ru (V.S.); 2 Institute of Solid State Chemistry and Mechanochemistry SB RAS, Kutateladze Str. 18, 630128 Novosibirsk, Russia; mikhailenko@solid.nsc.ru; 3 Budker Institute of Nuclear Physics SB RAS, Akad. Lavrentieva Ave. 11, 630090 Novosibirsk, Russia; mkor4@rambler.ru

**Keywords:** oxygen separation membranes, pyrochlores, bismuth cerate, bismuth titanate, oxygen mobility

## Abstract

Synthesis and study of materials based on bismuth cerates and titanates were carried out. Complex oxides Bi_1.6_Y_0.4_Ti_2_O_7_ were synthesized by the citrate route; Bi_2_Ce_2_O_7_ and Bi_1.6_Y_0.4_Ce_2_O_7_—by the Pechini method. The structural characteristics of materials after conventional sintering at 500–1300 °C were studied. It is demonstrated that the formation of a pure pyrochlore phase, Bi_1.6_Y_0.4_Ti_2_O_7_, occurs after high-temperature calcination. Complex oxides Bi_2_Ce_2_O_7_ and Bi_1.6_Y_0.4_Ce_2_O_7_ have a pyrochlore structure formed at low temperatures. Yttrium doping of bismuth cerate lowers the formation temperature of the pyrochlore phase. As a result of calcination at high temperatures, the pyrochlore phase transforms into the CeO_2_-like fluorite phase enriched by bismuth oxide. The influence of radiation-thermal sintering (RTS) conditions using e-beams was studied as well. In this case, dense ceramics are formed even at sufficiently low temperatures and short processing times. The transport characteristics of the obtained materials were studied. It has been shown that bismuth cerates have high oxygen conductivity. Conclusions are drawn about the oxygen diffusion mechanism for these systems. The materials studied are promising for use as oxygen-conducting layers in composite membranes.

## 1. Introduction

Oxides and solid solutions with the pyrochlore structure A_2_B_2_O_7_ (or A_2_B_2_O_6_O’, where A and B are rare earth or transition elements) attracted a lot of attention as materials for many applications such as oxygen [1,2,3,4] and hydrogen [5,6] separation membranes, solid oxide fuel cell/electrolyzer electrolytes [1,7,8,9] and electrodes [8,10,11], catalysts for various transformation processes [12,13], pigments [14,15], etc. [16,17,18]. The prospects of using pyrochlores in various electrochemical devices are provided by their high ionic or mixed ionic-electronic conductivity, depending on their composition and synthesis conditions [1,8,9,10,11,19,20].

The transport characteristics of pyrochlores are affected by their structural and defect features. In the real pyrochlore structure, various defects are present, including antistructural cation disordering:

(1) $$A_{A}^{\times }+B_{B}^{\times }⇄A_{B}^{\prime }+B_{A}^{•},$$

Frenkel anion disordering:

(2) $$O_{O}^{\times }⇄V_{O\;(48f)}^{••}+O_{i\;(8a)}^{\prime \prime },$$

partial ordering of the structure via association of defects, etc. [21,22,23,24,25]. In the A_2_B_2_O_6_O’ structure, O (48*f* Wyckoff positions) and O’ (8*a* Wyckoff positions) are non-equivalent and belong to two different sublattices (B_2_O_6_ and A_2_O’, respectively); however, while studying oxide ionic transport, these forms are sometimes undistinguishable, thus suggesting some kind of cooperative migration involving both O and O’ anions [20,26,27]. For some pyrochlores, the oxygen forms differing by their mobility are distinguishable; however, their fractions’ ratio differs from 6:1, hence probably supporting the abovementioned assumption or making evidence of other effects on the oxygen mobility such as different bonding energies of oxide anions in A–O–A, A–O–B and B–O–B chains [27,28]. The features of grain boundaries enhancing [28,29] or blocking [30] oxygen transport are reported as well.

As is known, the thermal instability of Bi_2_Ti_2_O_7_ at temperatures above 612 °C [31] is due to an unfavorable size factor (the ratio of bismuth and titanium cations), thus limiting the possibility of obtaining it in the form of dense ceramics for practical use. The stability of bismuth titanate pyrochlore can be achieved by replacing part of the bismuth atoms with atoms of other elements with a smaller ionic radius [32,33]. Doped bismuth titanates were previously studied and showed good transport properties with doping both A and B sites with various cations such as Co, Zn, etc., enhancing both ionic and total conductivity, which was probably associated with disordering of dopant cation distribution between A- and B-sublattices (Equation (1)) [20,26]. Hence, doped bismuth titanates can be assumed to be promising in electrochemical applications, such as components of permselective layers of oxygen separation membranes.

Bismuth cerates were previously studied as photocatalysts [13] and inorganic pigments [14,15]. Unfortunately, there is a lack of information on their transport properties; however, they probably possess good transport characteristics, especially oxygen mobility, due to the redox activity of Ce^4+^/Ce^3+^ cations and a high oxygen vacancy content [34,35,36]. Hence, they are of potential interest in electrochemical applications as well.

Obtaining functional ceramics (for solid oxide fuel cells and permselective membranes) with the required morphological and transport properties is a separate problem in materials science, where sintering is the most important procedure. With traditional thermal sintering in a furnace, long-term processing, and high temperatures are required to obtain ceramics with desired properties, such as gas tightness, homogeneous phase composition, etc. To solve this problem, radiation-thermal sintering (e-beam processing) is proposed [37,38,39,40,41]. Using the main advantages of radiation-thermal reactions—lowering the treatment temperature and a high reaction rate—will reduce the processing time while also significantly reducing internal thermal stresses. This technique was successfully applied for sintering functional layers of solid oxide fuel cells (thin layers of electrolytes such as Y or Sc + Ce -doped zirconia, Gd-doped ceria, etc. on anode substrates, nanocomposite cathode layers such as LSM–ScCeSZ, PrNiCoO-GDC, etc.) and asymmetrically supported oxygen separation membranes (thin and dense permselective layers of mixed ionic-electronic conductors comprised of complex oxides with perovskite, fluorite, spinel, etc. structures or their nanocomposites, such as LFN-GDC, LFC-GDC, La_0.3_Bi_0.7_MnO_x_–Bi_1.5_Y_0.3_Sm_0.2_O_3_, etc.) using unique equipment of the Budker Institute of Nuclear Physics [37,38,39,40,41]. Disordering of nanodomains by electron beams leads to their easy sintering at moderate temperatures without increasing their sizes. This results in a developed network of domain boundaries, which, for nanocomposites [37,38,39,40,41] or even some complex oxides (such as molybdates of lanthanoids [42], etc.), provide fast oxygen diffusion channels described by the so-called 2D model of oxygen diffusion. For solid oxide fuel cells and asymmetric oxygen separation membranes on metallic substrates, due to the decreased temperature and duration of sintering as compared with conventional sintering methods, such negative phenomena as a variation of functional layer phase composition, cracking, and damage to metallic substrates were prevented. This also allowed for maximum power densities of thin-film solid oxide fuel cells with nanocomposite perovskite-fluorite cathode layers operating on wet H_2_ as fuel and air as oxidant up to 500 mV/cm^2^ at 700 °C, which is promising for practical application. For an asymmetric membrane comprised of LaBiMnO-YSmBiO layers supported on a binary Ni-Al foam substrate, the oxygen flux under the air/He gradient was up to 5 mL O_2_ at 950 °C, which is really high [41]. However, at such high temperatures, Bi can be evaporated from these oxides; hence, to deal with this problem, doped Bi cerates and titanates known to be more chemically stable were studied in this work.

This work aimed at studying the structural and oxide ionic transport properties of bismuth titanates and cerates, including Y-doped ones, and the effects of such processing as radiation-thermal sintering for pyrochlores. Its realization will provide the basis for manufacturing materials for solid oxide fuel cells or oxygen-conducting membranes. The effect of doping with Y on the structural stability and phase composition of these materials was studied. The sinterability of samples was investigated in order to check the adaptability of these materials to obtain gas-tight ceramics for electrochemical applications, including radiation-thermal sintering by e-beams. The oxygen transport properties were studied by the temperature-programmed isotope exchange of oxygen with C^18^O_2_ in the flow reactor to acquire data on the oxygen diffusivity required for these applications.

## 2. Materials and Methods

Bi_2_Ce_2_O_7_ and Bi_1.6_Y_0.4_Ce_2_O_7_ were synthesized via the modified Pechini technique, as described elsewhere [43]. For the Bi_1.6_Y_0.4_Ti_2_O_7_ sample synthesized by the citrate method, corresponding salts in the required ratios were added to a solution of citric acid in water (1;5 mole ratio), while the Me:citric acid ratio was equal to 1:2. Bi(NO_3_)_3_·5H_2_O (>99%), Ce(NO_3_)_3_·6H_2_O (>99%), Ti (OCH(CH_3_)_2_)_4_ (>99%), and Y(NO_3_)_3_·6H_2_O (>99%) were used as initial reagents. The xerogels obtained were dried in a drying oven at 110 °C for 12 h, then calcined at 500 °C for 3 h. As-prepared powders were pressed into pellets and sintered at 700 °C for 3 h, at 900 °C for 3 h, at 1100 °C for 10 h, and at 1300 °C for 8 h using conventional sintering.

Radiation-thermal sintering was carried out using an accelerator, ILU-6 (BINP SB RAS, Russia). Electron pulses with 2.4 MeV energy, 328 mA pulse beam current, a pulse duration ~600 s, a narrow scan, and up to 25 Hz pulse frequency were used. The temperature of the samples was controlled using a Pt-Pt-Rh thermocouple and FildPoint (National Instruments, USA) controlling module. Power adjustment was carried out by changing the frequency of pulses. The heating rate was 50 C min^−1^, and after achieving 1100 °C, samples were sintered for 30 min.

X-ray diffraction (XRD) studies were performed using a Bruker D8 Advance diffractometer with the Lynx-Eye detector using CuKα radiation. XRD patterns were recorded in the 2*θ* range of 15–95° with a step of 0.05°. Rietveld refinement for quantitative analysis and calculation of lattice parameters was carried out using the software package Topas V.4.2.

Infrared (IR) spectra (4000–250 cm^−1^, 32 scans, resolution 4 cm^−1^) were acquired using a Cary 660 (Agilent Technologies, Santa Clara, CA, USA) spectrometer with the GladiATR PIKE Technologies console.

Scanning electron microscopy studies were carried out using a dual-beam scanning electron microscope, the Tescan Solaris FE/SEM (Tescan, Brno, Czech Republic). The experiments were performed in the secondary electron mode at an accelerating voltage of 20 kV.

Oxygen mobility and surface reactivity were studied by the temperature-programmed isotope exchange (TPIE) of oxygen with C^18^O_2_ in the flow reactor. Samples (0.25–0.5 mm fraction) were loaded into quartz tube reactors (with an inner diameter of 3 mm). Pretreatment was carried out in He + 1% O_2_ flow (25 mL min^−1^) at 700 °C for 30 min. The isotope exchange was carried out in He + 1% C^18^O_2_ + 1% Ar flow (25 mL min^−1^) while heating from 50 to 800 °C with a constant ramp of 5 °C min^−1^. ^18^O atomic fraction (*α*) and C^16^O^18^O atomic fraction (*f*_16–18_) responses were analyzed in order to estimate isotope exchange kinetic parameters.

## 3. Results and Discussion

### 3.1. Structural Features

Compositions and lattice parameters of all prepared samples are presented in Table 1.

**Figure 1:**
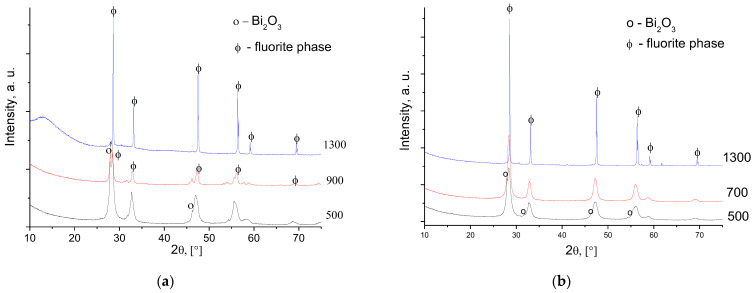
XRD patterns of Bi_2_Ce_2_O_7_ (**a**) and Bi_1.6_Y_0.4_Ce_2_O_7_ (**b**) obtained by conventional sintering at various temperatures.

According to the XRD data, for all cerate samples after calcination at 500–700 °C, there is an admixture of bismuth oxide (Figure 1). For bismuth cerates’ samples, the cubic fluorite phase forms after sintering at low temperatures. Metastable bismuth cerate is proposed to form the solid solution in the ceria-yttria complex oxide (Figure 1b). Similar behavior was observed for the undoped Bi cerate: metastable Bi cerate forms after synthesis, and at further sintering, it decomposes into the oxide mixture, followed by forming the solid solution (Figure 1a). The introduction of Y^3+^ into Bi_2_Ce_2_O_7_ decreases the lattice constant from 5.413 Å to 5.407 Å, thus evidencing substitution of ions in the lattice with contraction as expected, since the ionic radius of Y^3+^ (r = 1.01 Å, CN = 8) is smaller than that of Bi^3+^ (r = 1.18 Å, CN = 8) (Table 1). With increasing the temperature of sintering, there is a decrease in the lattice constants for the yttrium-doped bismuth cerates, suggesting a higher disordering of their structure. After sintering at 1300 °C, the XRD pattern for Bi_1.6_Y_0.4_Ce_2_O_7_ contains peaks attributed to the disordered CeO_2_ fluorite phase, which is visible according to the peaks’ shift.

**Figure 2:**
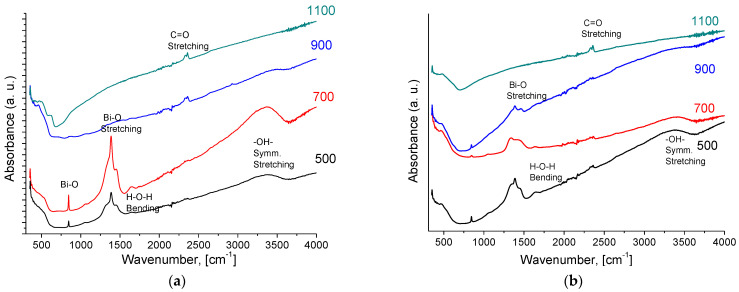
IR spectra of Bi_2_Ce_2_O_7_ (**a**) and Bi_1.6_Y_0.4_Ce_2_O_7_ (**b**) obtained by conventional sintering at various temperatures.

According to IR spectroscopy data for both Bi cerate samples, the bands at 1631 cm^−1^ and 3401 cm^−1^ observed in IR spectra correspond to –OH symmetric vibrations appearing due to water adsorption (Figure 2). The increasing temperature of sintering leads to the removal of chemisorbed water from the surface. The most intense band at 1383 cm^−1^ corresponds to Bi–O bond vibrations [12,44,45]. The peak corresponding to the C–O functional group at 2367 cm^−1^ is shown in Figure 2 [33]. The absorption band that appears at wavelengths below 500 cm^−1^ presents the stretching vibration in the structure of the cerium oxide (Ce-O-Bi) mode [46].

**Figure 3:**
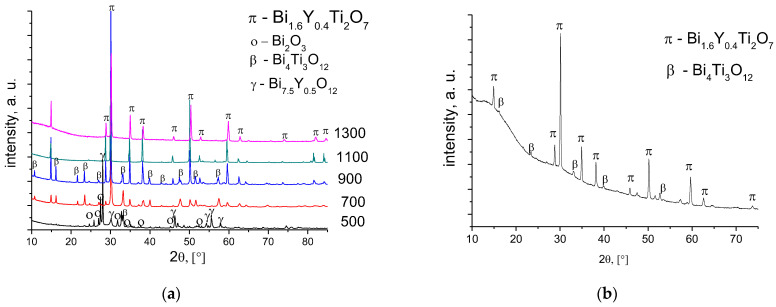
XRD patterns of Bi_1.6_Y_0.4_Ti_2_O_7_ sintered at various temperatures using conventional sintering (**a**) and radiation-thermal sintering at 1100 °C (**b**).

The Bi_1.6_Y_0.4_Ti_2_O_7_ samples after sintering at 500 and 900 °C are not single-phased (Figure 3a). According to the XRD data, for titanate sample as in the case of cerates after calcination at 500–700 °C, there is an admixture of bismuth oxide (Figure 3a). Bismuth titanates, along with the pyrochlore phase corresponding to the cubic Bi_1.74_Ti_2_O_6.624_ [PDF 089-4732] phase, contain admixtures of the tetragonal Bi_4_Ti_3_O_12_ [047-0398] and tetragonal Bi_2_O_3_ [PDF 071-0465] phases.

The Y-doped Bi titanate sintered at 1100–1300 °C contains no admixtures. From the point of view of the phase composition, the RTS conditions used in this work did not allow us to obtain a single-phase sample (Figure 3b). The content of the impurity phase, Bi_4_Ti_3_O_12,_ was 8%.

**Figure 4:**
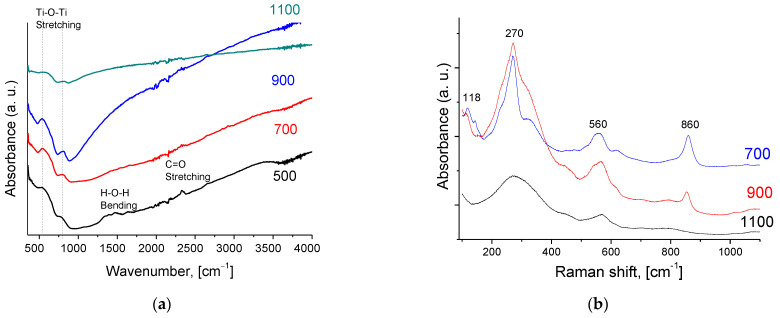
IR (**a**) and RAMAN (**b**) spectra of Bi_1.6_Y_0.4_Ti_2_O_7_ samples sintered at various temperatures using conventional sintering.

As in the case of bismuth cerates’ samples, the bands observed at 1631 cm^−1^ and 3401 cm^−1^ are explained by –OH symmetric vibrations appearing due to water adsorption (Figure 4a). The characteristic feature of the IR spectra of pyrochlores is the presence of an intense band in the range of 400–600 cm^−1^ [12].

RAMAN spectra for Bi_1.6_Y_0.4_Ti_2_O_7_ samples are given in Figure 4b. Typical vibration modes present in the spectra demonstrate the formation of the pyrochlore phase only after high-temperature calcination, in good agreement with the XRD data [12,47]. According to the XRD data, the Bi_4_Ti_3_O_12_ phase is present for doped bismuth titanate after calcination at 700–900 °C. In the low-frequency region of the spectrum, the lines at 50–150 cm^−1^ correspond to Bi oscillations relative to oxygen octahedra. Modes in the frequency range of 200–400 cm^−1^ correspond to deformation vibrations of O–Ti–O bonds, and high-frequency bands in the range of 500–850 cm^−1^ correspond to valence vibrations. There is also a band corresponding to the full-symmetric valence oscillation of O–Ti-O bonds of octahedra with a frequency of 843 cm^−1^.

**Figure 5:**
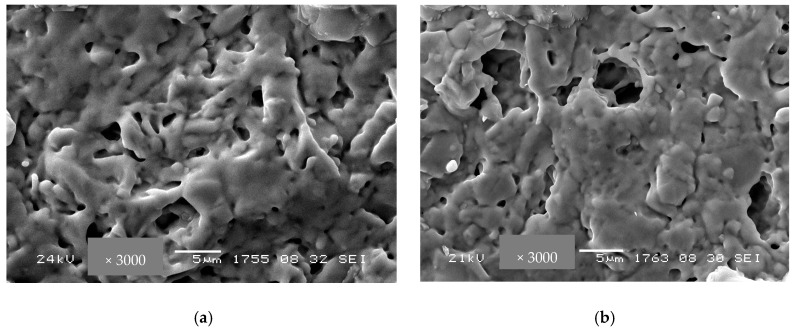
SEM micrographs of Bi_2_Ce_2_O_7_ (**a**) and Bi_1.6_Y_0.4_Ce_2_O_7_ (**b**) obtained by conventional sintering at 1100 °C.

**Figure 6:**
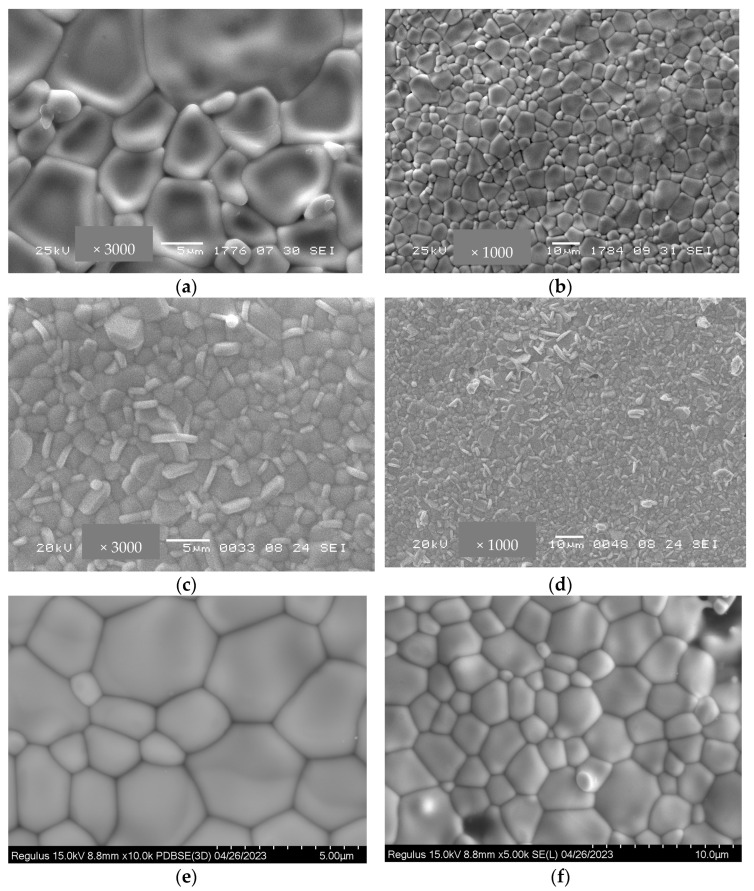
SEM micrographs of Bi_1.6_Y_0.4_Ti_2_O_7_ obtained by conventional sintering at 1300 °C for 10 h (**a**,**b**), at 1100 °C for 10 h (**c**,**d**), and radiation-thermal sintering at 1100 °C for 30 min (**e**,**f**).

Figure 5 demonstrates SEM micrographs of Bi_2_Ce_2_O_7_ and Bi_1.6_Y_0.4_Ce_2_O_7_ after conventional sintering at 1100 °C. For both samples, pores have an irregular shape, with their size varying from a micrometer to a few micrometers. Conventionally sintered bismuth titanates’ samples have larger particles compared to the samples after RTS at 1100 °C (Figure 6). A similar tendency was demonstrated in [37] for lanthanide tungstates and molybdates. Using the traditional sintering of bismuth titanate at 1100 °C, the average grain size ranges from 1 to 10 microns. The different morphology of the particles visible here is due to the admixture of the Bi_4_Ti_3_O_12_ phase. Sintering at this temperature for 10 h was not sufficient since the presence of pores is visible (Figure 6c,d). The porosity and average pore size values are given in Table 2. Figure 6e,f, showing SEM images of RTS Bi_1.6_Y_0.4_Ti_2_O_7_, do not contain microcracks and pores, which demonstrates the optimal sintering conditions. The difference in particle size is apparently caused by the effects of the sintering technique (conventional or radiation-thermal), sintering temperature, and duration, which affect the particles’ aggregation and growth. Hence, it was demonstrated that RTS allows for carrying out sintering processes for shorter times and at lower temperatures compared to those for conventional sintering. Such a difference in sintering temperature and duration required to obtain the desired gas-tightness for radiation–thermal sintering and conventional sintering is apparently related to the dissipation of radiation energy in heterogeneous structures, thermal-diffusional stimulation of mass transfer, and other features of the RTS process [37].

### 3.2. Oxygen Transport Features

**Figure 7:**
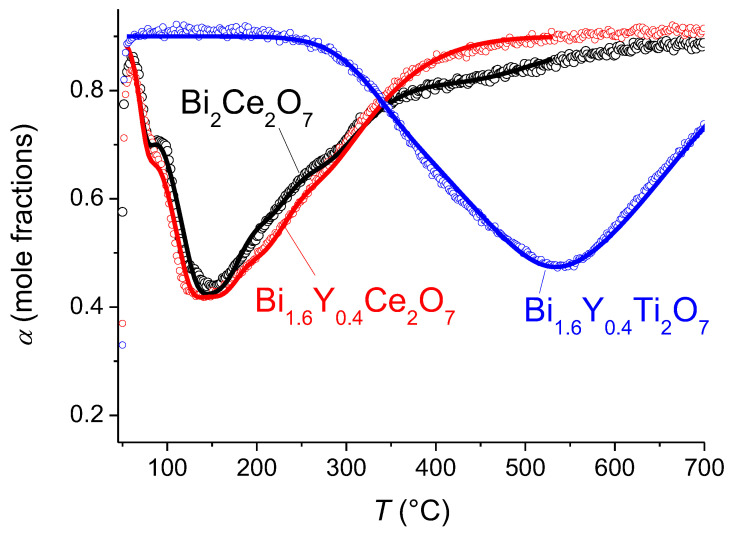
Temperature-programmed isotope exchange of oxygen with C^18^O_2_ in a flow reactor for bismuth cerate and titanate samples sintered at 700 °C. Points—experiment, lines—modeling.

**Figure 8:**
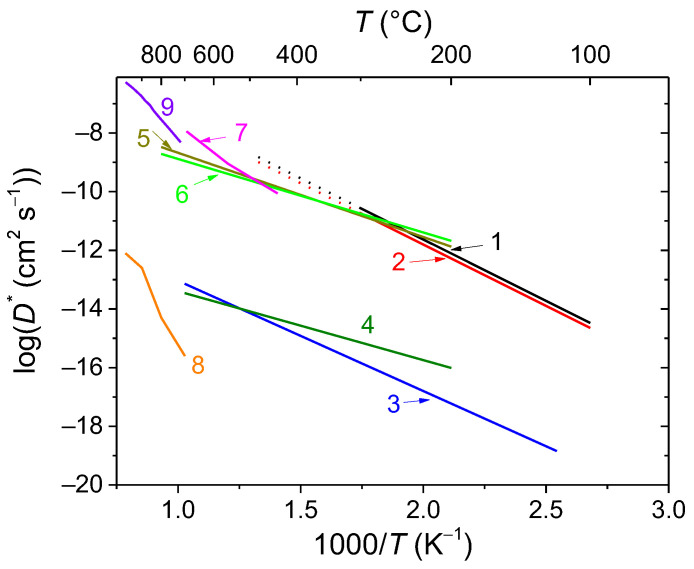
Arrhenius plots for oxygen tracer diffusion coefficients acquired by TPIE data modeling for Bi_2_Ce_2_O_7_ (1), Bi_1.6_Y_0.4_Ce_2_O_7_ (2), and Bi_1.6_Y_0.4_Ti_2_O_7_ (3) samples sintered at 700 °C compared to other oxide materials: Bi_1.6_Sc_0.2_Ti_2_O_7−δ_ (4) [26], Bi_1.6_Mg_0.2_Ti_2_O_7−δ_ (5) [26], Bi_1.6_Zn_0.2_Ti_2_O_7−δ_ (6) [20], Zr_0.84_Y_0.16_O_1.92_ (7) [49], La_0.8_Sr_0.2_MnO_3−δ_ (8) [50], La_0.5_Sr_0.5_Fe_0.7_Co_0.3_O_3−δ_ (9) [51].

According to the TPIE with C^18^O_2_ data for the bismuth cerate sample, a few peaks in the TPIE curve are visible, providing evidence of strong nonuniformity of oxygen mobility (Figure 7). According to the numeric analysis, the first peak (70 °C) is related to the substitution of the surface oxygen and is characterized by a surface exchange effective activation energy of 150 kJ mol^−1^. The most clearly expressed peak (150 °C) is determined by fast oxygen diffusion, which involves about 1/3 of the total oxygen content. This is probably oxygen-bound cerium. The rate of substitution of the rest of the oxygen is characterized by less-expressed peaks. For the description, the model including a single diffusion channel across the most mobile oxygen of Ce–O–Ce chains with subsequent exchange with neighboring more strongly bound oxide anions in the lattice was used [48]. The mean integral exchange coefficient (*β*) is 0.012 min^−1^ for bismuth cerate. The calculated parameters of the isotope exchange are given in Table 3. The Arrhenius plots of the oxygen tracer diffusion coefficient are given in Figure 8.

For the Y-doped Bi cerate sample, a similar behavior of isotope substitution dynamics is observed. The same model as that for the undoped sample [48] was used. The difference in the diffusion rate via the Ce–O–Ce channel is insignificant; however, the exchange with other forms of oxygen is significantly faster. The mean integral exchange coefficient (*β*) is 0.017 min^−1^ for Y-doped bismuth cerate (Table 3).

For Y-doped Bi titanate, the oxygen substitution rate is significantly lower than that for Bi cerates. The isotope propagation rate is described by the uniform diffusion model with an identical oxygen tracer diffusion coefficient within the entire volume.

Such a difference in the oxygen mobility of doped Bi titanate and Bi cerates is probably related to the content of oxygen vacancies participating in oxide ions’ migration via M–O–M channels. The redox activity of Ce^4+^/Ce^3+^ cations [34,35,36] is probably responsible for the higher oxygen vacancies’ content for Bi cerates compared to that for Bi titanates. The possible evidence of this is the frequency and intensity of bands corresponding to the H–O–H bending in IR spectra for Bi cerates and titanates (Figure 2 and Figure 4), since these bands appear due to water adsorption with the participation of oxygen vacancies [33]:

(3) $$H_{2}O\;+\;V_{O\;}^{••}⇄2H_{}^{•}+O_{O\;}^{\times },$$

(4) $$H_{2}O\;+\;O_{O\;}^{\times }+V_{O\;}^{••}⇄2OH_{O\;}^{•}.$$

While compared with other Bi titanate-based pyrochlores, the oxygen tracer diffusion coefficient values of Y-doped Bi titanate are slightly lower than those for Sc-doped Bi titanate and significantly lower than those for Mg- and Zn-doped Bi titanates (Figure 8, curves 4–6) previously studied by authors [18,24]. This is probably due to the difference in cation size and charge as well as A-site stoichiometry (the samples from works [20,26] are A-site deficient) and, hence, oxygen vacancy content and space in the lattice for oxygen migration.

It is difficult to compare the Bi cerate-based pyrochlores studied in this work with similar materials since there is a lack of information on the oxygen mobility of Bi cerates in the literature. However, it is comparable to or higher than that for Mg- and Zn-doped Bi titanates (Figure 8, curves 4–6) [20,26], exceeding that for commonly used ionic-conducting and MIEC materials for oxygen separation membrane components such as YSZ, LSM, and LSFC (Figure 8, curves 7–9) [49,50,51].

As mentioned in the Introduction, pyrochlore-like oxide materials can be utilized in catalytic reactors based on oxygen [1,2] and hydrogen [5,6] separation membranes for hydrogen and syngas production via fuel transformation reactions. For such reactors based on oxygen separation membranes, a high oxygen mobility (along with a high mixed ionic-electronic conductivity) allows for high oxygen permeation fluxes across the membrane from the air side onto the fuel side, thereby providing efficient performance in fuel transformation reactions [1,2,52,53,54]. For catalytic membrane reactors based on hydrogen separation membranes, high oxygen mobility is also desirable for the membrane materials. This is due to some proton transport mechanisms being mediated by oxygen transport, such as the vehicle mechanism [52,55,56], as well as the vehicular transport of structurally bound water proposed for some oxides [57,58]. Moreover, the presence of oxide-ionic conductivity in the hydrogen separation membrane allows for additional hydrogen yield due to the water splitting reaction while humidifying the purge side feed [59,60]. Finally, the application of triple (protonic—oxide-ionic—electronic) conducting materials in membrane reactors allows for enhance the reactor performance in various catalytic reactions and to improve gas separation characteristics due to coupled transport of electrons/holes, oxide anions/vacancies, and protons, forcing any of these species to be transported due to their chemical potential gradient [61,62,63].

Hence, undoped and Y-doped Bi cerates studied in the current work possessing a high oxygen mobility (Table 3, Figure 8) meet the criteria for use in oxygen separation membrane-based reactors for hydrogen and syngas production [1,2,52,53,54]. The Y-doped Bi titanate involved in this work possesses moderate oxygen mobility (Table 2, Figure 8) and may be used in oxygen separation membranes as well; however, in order to achieve a high oxygen permeation flux, additional modification and/or use as a component of composite membranes can be recommended. It is to be noted that the materials studied can probably be used in hydrogen separation membrane-based reactors as well due to their oxygen transport properties, which enable them to predict good proton transport properties [5,47,56]. However, since these materials are initially intended for potential application in oxygen separation membranes, investigating the proton transport properties of these Bi cerates and titanates is outside the scope of this work and requires a separate study.

## 4. Conclusions

Complex oxides Bi_1.6_Ti_0.4_Ce_2_O_7_, Bi_2_Ce_2_O_7_, and Bi_1.6_Y_0.4_Ce_2_O_7_ were synthesized using Pechini and citrate methods, and the structural characteristics after calcination using conventional and e-beam sintering were studied. It was shown that the formation of the pure pyrochlore phase Bi_1.6_Y_0.4_Ti_2_O_7_ occurs after high-temperature calcination at 1100 °C. In addition, the complex oxides Bi_2_Ce_2_O_7_ and Bi_1.6_Y_0.4_Ce_2_O_7_ have a fluorite structure with negligible amounts of Bi_2_O_3_ formed at low temperatures. As a result of calcination at high temperatures, the pyrochlore phase turns into a fluorite CeO_2_ phase enriched with bismuth oxide. RTS of bismuth titanate made it possible to obtain fine-grained ceramics in a minimum processing time and at a much lower temperature. The transport characteristics of pyrochlore samples were studied. It has been shown that bismuth cerates have high oxygen conductivity. Conclusions are drawn about the mechanism of oxygen diffusion in these systems. The obtained materials can potentially be used as oxygen-conducting layers of catalytic membranes.

## Figures and Tables

**Table 1:** Structural properties of prepared pyrochlores.

№	Composition	T_calcin._, °C	Phase	Lattice Parameter, Å	Crystallite Size, Å
1	Bi_2_Ce_2_O_7_	500	CeO_2_	5.463 (1)	100
			Bi_2_O_3_	-	200
		900	CeO_2_	5.435 (1)	260
			Bi_2_O_3_	-	340
		1300	CeO_2_	5.413 (1)	>1500
2	Bi_1.6_Y_0.4_Ce_2_O_7_	500	Bi_1.2_Y_0.8_O_3_ (CeO_2_) Bi_2_O_3_	5.441 (1)	85
		700	CeO_2_	5.437 (1)	130
		1300	CeO_2_	5.407 (1)	>1500
3	Bi_1.6_Y_0.4_Ti_2_O_7_	500	30% Bi_4_Ti_3_O_12_	a = 5.5 (1), c = 5.4 (1), c = 32.94 (5)	>1500
			40% Bi_7.5_Y_0.5_O_12_	a = b = 7.725 (1), c = 5.632 (1)	150
			30% Bi_2_O_3_	-	1000
		700	60% Bi_4_Ti_3_O_12_ 40% Bi_1.74_Ti_2_O_6.624_ (pyrochlore)	a = 5.385 (1), c = 5.409 (1), c = 32.820 (6) a = 10.277 (2)	640 600
		900	70% Bi_1.74_Ti_2_O_6.624_	a = 10.289 (1)	>1500
			30% Bi_4_Ti_3_O_12_	a = 5.430 (1), c = 5.402 (1), c = 32.895 (4)	>1500
		1100	Bi_1.74_Ti_2_O_6.624_	a = 10.310 (1)	>1500
		1100 After RTS	92% Bi_1.74_Ti_2_O_6.624_	a = 10.273 (2)	>1500
			8% Bi_4_Ti_3_O_12_	a = 5.422 (3), c = 5.392 (3), c = 32.80 (2)	900

**Table 2:** The pore parameters for Bi_2−x_Y_x_M_2_O_7_ (M = Ce, Ti) samples.

Sample	Sintering Temperature (°C)	Sintering Technique	Porosity (%)	Mean Pore Size (μm)
Bi_2_Ce_2_O_7_	1100	Conventional	7	1.2
Bi_1.6_Y_0.4_Ce_2_O_7_	1100	Conventional	13	1.9
Bi_1.6_Y_0.4_Ti_2_O_7_	1100	Conventional	3	0.83
		Radiation-thermal	1	0.25
	1300	Conventional	2	0.76

**Table 3:** The values of surface heteroexchange rate (*R*), tracer diffusion coefficient normalized by mean diffusion pathway (D*/*L*^2^), bulk oxygen exchange coefficient (*β*) at 120 °C, and their effective activation energies (*E_R_*, *E_D_*, *E_β_*, respectively) calculated according to TPIE data modeling.

Sample	*R* (min^−1^)	*E_R_* (kJ mol^−1^)	D*/*L*^2^ (min^−1^)	*E_D_* (kJ mol^−1^)	*β* (min^−1^)	*E_β_* (kJ mol^−1^)
Bi_2_Ce_2_O_7_	1.8 × 10^2^	150	0.07 (32%)	80	0.012	80
Bi_1.6_Y_0.4_Ce_2_O_7_	1.8 × 10^2^	150	0.11 (34%)	80	0.017	80
Bi_1.6_Y_0.4_Ti_2_O_7_	1 × 10^−5^	100	4.7 × 10^−7^ (100%)	72		

Note: * means that this is a tracer diffusion coefficient related to the isotope tracer ^18^O. It is related to the oxygen self-diffusion coefficient via a correlation factor f corresponding to the counterflows of isotopes ^16^O and ^18^O within the sample bulk (D* = f × DO, f ≈ 0.5, …, 1). A mean diffusion pathway has a meaning of an average particle size. A surface heteroexchange rate (R) characterizes the rate of exchange of oxygen between CO_2_ in the gas phase and the oxide on its surface.

## Data Availability

Not applicable.
